# The use of anti-GITR antibody treatment in a murine model of glioblastoma multiforme

**DOI:** 10.1186/2051-1426-3-S2-P236

**Published:** 2015-11-04

**Authors:** Jason Miska, Alan L Chang, Aida Rashidi, Mahua Dey, Yu Han, Lingjiao Zhang, Irina V Balyasnikova, Atique U Ahmed, Maciej S Lesniak

**Affiliations:** 1 The University of Chicago, Chicago, IL, USA; 2 University of Chicago, Chicago, IL, USA

## 

Glioblastoma multiforme (GBM), is the most common adult brain tumor and despite many new therapies, remains largely incurable. Regulatory T cells (Treg) are highly immunosuppressive cells that accumulate within glioma microenvironment, and reduction of their function or trafficking has been previously shown to enhance survival in pre-clinical models of GBM. One of the principal mechanisms of Treg suppression *in vitro* is that they must have direct contact with their targets; a modality recently appreciated *in vivo*. It is thought that Tregs utilize the cytotoxic proteins perforin and granzyme to directly lyse target immune cells. While this might be a major effector in preventing the anti-tumor response, little has been studied on the importance this pathway is in the context of glioma.

An agonistic antibody against GITR (glucocorticoid induced TNF family receptor) has been demonstrated to negatively influence Tregs in a number of animal models. However, the role of this therapy in GBM has not yet been explored. We hypothesized that anti-GITR treatment suppresses granzyme expression by Tregs, which results in stronger anti-tumor immune responses against glioma.

Using the GL261 murine model of GBM, 4 × 10^5^ cells were stereotactically injected in the right hemisphere of 6-8 week old mice. Mice received anti-GITR or its isotype control directly into the tumor site (2ug) or *i.p.* (500ug) at both 9 and 14 days post implantation Mice were separately assessed for survival, and flow cytometric analyses were performed to determine the influence of the antibody on immune response in the brain.

Analysis revealed that many Tregs specifically within tumor tissue express granzyme B, which increases over time. Animals treated with anti-GITR antibody caused a significant increase in animal survival only when injected directly into the tumor site, while peripheral treatment only had a modest effect. Antibody treatment caused a significant decrease in granzyme B^+^ Tregs with peripheral treatment. However, with intratumoral treatment, we saw both a decrease in granzyme expression and Treg number.

Our data suggests that within the glioma microenvironment, Tregs gain the ability to produce granzyme B, which can be suppressed by administration of anti-GITR antibody treatment, but this mechanism is not alone sufficient to enhance animal survival. However, intratumoral anti-GITR treatment dramatically reduced Tregs and their granzyme B expression, suggesting another mechanism in this therapeutic modality. This study shows that the site of anti-GITR administration could be critical in its efficacy when taken into the clinic.

